# Preferences heterogeneity of health care utilization of community residents in China: a stated preference discrete choice experiment

**DOI:** 10.1186/s12913-020-05134-4

**Published:** 2020-05-18

**Authors:** Ming-zhu Jiang, Qiang Fu, Ju-yang Xiong, Xiang-lin Li, Er-ping Jia, Ying-ying Peng, Xiao Shen

**Affiliations:** 1 School of Medicine and Health Management, Tongji Medical College, Huazhong University of Science and Technology, Wuhan, 430030 Hubei China; 2 Department of Epidemiology and Biostatistics, College for Public Health and Social Justice, Saint Louis University, St. Louis, MO USA

**Keywords:** Discrete choice experiment, Preference heterogeneity, Healthcare utilization

## Abstract

**Background:**

To tackle the issue with the low usage of primary healthcare service in China, it is essential to align resource distribution with the preferences of the community residents. There are few academic researches for describing residents’ perceived characteristics of healthcare services in China. This study aims to investigate the preferences of healthcare services utilization in community residents and explore the heterogeneity. The findings will be useful for the policy makers to take targeted measures to tailor the provision of healthcare services.

**Methods:**

The face-to-face interviews and surveys were conducted to elicit four key attributes (care provider; mode of services; cost; travel time) of the preference from community residents for healthcare utilization. A rational test was presented first to confirm the consistency, and then 16 pairs of choice tasks with 12 sociodemographic items were given to the respondents. Two hypothetical options for each set, without an opt-out option, were presented in each choice task. The latent class analysis (LCA) was used to analyse the data.

**Results:**

Two thousand one hundred sixty respondents from 36 communities in 6 cities were recruited for our study. 2019 (93.47%) respondents completed valid discrete choice experiment (DCE) questionnaires. The LCA results suggested that four groups of similar preferences were identified. The first group (27.29%) labelled as “Comprehensive consideration” had an even preference of all four attributes. The second group (37.79%) labelled as “Price-driven” preferred low-price healthcare services. The third group labelled as “Near distance” showed a clear preference for seeking healthcare services nearby. The fourth group (34.18%) labelled as “Quality seeker” preferred the healthcare service provided by experts. Willingness to pay (WTP) results showed that people were willing to accept CNY202.12($29.37) for Traditional Chinese Medicine (TCM) services and willing to pay CNY604.31($87.81) for the service provided by experts.

**Conclusions:**

Our study qualitatively measures the distinct preferences for healthcare utilization in community residents in China. The results suggest that the care provider, mode of services, travel time and cost should be considered in priority setting decisions. The study, however, reveals substantial disagreement in opinion of TCM between different population subgroups.

## Background

China has committed itself to promoting health system reform since 2009 and this is a large-scale, long-term endeavour. The ratio of total expenditure on health to gross domestic product (GDP) increased from 5.03% in 2009 to 6.36% in 2017. In the same period, the government’s expenditure on health increased by CNY10–15 billion ($14.55–21.83 billion) per year [1].

Indeed, government subsidies to primary healthcare facilities (including community health centers and stations at community and district levels in urban area, county and township health centers and village clinics at county, township, and village levels in rural areas) have increased substantially: from 2009 to 2017, subsidies as a proportion of total primary healthcare facilities revenue increased from 12.3 to 32.5% [1].

However, despite the increase in funding, the proportion of outpatient visits at primary healthcare facilities decreased relative to that at tertiary hospitals, and the proportion of hospitalisations at tertiary hospitals increased. From 2012 to 2016, the proportion of outpatient services provided by hospitals among all outpatient services continued to increase by 5.1% (from 30.8 to 35.9%). Public hospital admissions grew by 11.5% (from 61.7 to 73.1%), while the proportion of services provided by primary healthcare facilities decreased by 7% during the same period [2], suggesting that primary care providers were not effective gatekeepers.

There might be multiple causes leading to the current low usage of primary healthcare services, including historic and institutional factors [3]. First, compared with tertiary hospitals, the supply capacity of primary healthcare facilities is insufficient. Approximately 58% of resources were concentrated at tertiary hospitals, while only 18% at primary healthcare facilities [4]. Such resource allocation is inefficient, costly, and cannot fully meet the residents’ healthcare needs [5]. Secondly, subpar medical quality and diagnostic inaccuracies exist in primary care facilities. A study revealed that only 38 and 28% of correct treatments in township health centres and village clinics were provided for patients with incognito tuberculosis, respectively [6]. A national survey showed that primary care facilities in China prescribed 52.9% of their patients with antibiotics, whereas only 39.4% of the cases required such treatment [7]. The services provided in primary healthcare facilities in China are often considered inappropriate or of poor quality, and residents tend to seek more specialised consultations and higher quality services in tertiary hospitals in lieu of primary healthcare institutions [8].

China has reached a consensus that a good primary care system is essential for the overall wellbeing of the population. Compared to the current hospital-centred and fragmented delivery system, a shift to the primary-care-centred and integrated delivery system is more effective in response to the challenges of urbanization, industrialization, population ageing, and noncommunicable chronic disease [9].

However, many supporting policies have not shown a significant effect on improving the utilization of healthcare services in primary health facilities [10, 11]. Moreover, medical expenses have increased more rapidly since 2009. In tertiary hospitals, the outpatient fee per time increased by 15.5% from 2005 to 2008 and 21.7% from 2010 to 2013 [1]. Residents would prefer to spend more time, money and energy in tertiary hospitals rather than primary healthcare facilities for health, such healthcare seeking behaviour is deemed as costly and ineffective and hinders enhancement of service efficiency and improvements on user experience [8].

Understanding resident’s preference can bridge the information gap between the healthcare demander and the health policy decision-maker [12]. A series of factors are reported to influence health services utilization [13]. The impacts of these factors are not necessarily homogenous, but may be conditional on individual and contextual factors [14]. Further scientific evidence focused on how factors affect residents’ healthcare utilization is essential to encourage the usage of primary healthcare facilities in China.

Furthermore, understanding individual participation and treatment preferences can greatly improve shared decision making and their adherence. The success to measure residents’ preference may conducive to the medical system reform progress and resolve the needless expenditure [15]. Once considerations of healthcare values and priorities are clearly determined, then health policy decision-makers can establish a better quality of health care system more effectively [16].

Growing evidence suggests that people make active usage choices in seeking care and highlights the need to understand resident’s preference for choosing health services [17, 18]. Many countries aim to apply residents’ choices to support health service design. In UK, The London Patient Choice Project (LPCP) is established to improve choices for patients who are clinically eligible for treatment and who have been waiting for treatment at a National Health Service London hospital beyond some target waiting time. As the target waiting time approaches, patients are given an opportunity to choose from a range of alternative providers who have the capacity to offer health services in a timely manner [19].

Chinese scholars have identified the factors that could affect health care utilization. Shanshan Xu found that securing health insurance could increase the preference for in-patient service [20]. Xiaoqing Gan suggested that adjusting medical care reimbursement rates might contribute to meeting patients’ needs [21]. Min Wei reported that factors such as operation level, nursing quality and equipment conditions could affect residents’ choice [22]. Thus, it can be seen that there were few academic researches for describing residents’ perceived characteristics of healthcare services, which hence brings a crucial need to explore preferences in depth. Residents’ preferences are insufficiently included in China’s policy development process, and the key to reshaping China’s health care systems is to understand residents’ preferences. Taking the public’s demand for care as the basis of medical policy will help to enhance the value of medical services and promote rational allocation of health resources at the primary-care level.

As a non-market resource value evaluation technique, the DCE is an effective method to investigate the preference [23, 24]. DCE is grounded in Lancaster’s Economic Theory of Value [25] and McFadden’s Random Utility Theory [26]. In DCE’s design, an individual is asked to choose between different alternatives, which are described by attributes and levels [27]. Choices are then analysed to deconstruct respondents’ preferences based on the alternatives that they have chosen. When the cost is included as an attribute, marginal utility estimated from the DCE model can be converted into the willingness to pay for changes in the levels of other attributes [28]. The method of DCE has been widely used to evaluate different healthcare interventions around the world [29, 30]. Within the context of the health care system in China, our specific aims are to explore heterogeneity in community residents’ preferences for healthcare utilization and offer recommendations to promote service improvements and countermeasures. The findings will be useful in the distribution of healthcare resources and informing decision-makers on how to tailor the provision of healthcare services.

## Methods

### Selection of choice attributes and levels for the DCE

The performance of DCE is well described in the literature, and the literature provides clear guidance on how a DCE study should be done [31, 32]. A standard DCE consists of five steps: (1) selecting the attributes and levels to be included in the experiment, (2) developing the questionnaire and actual choice tasks presented to respondents, (3) producing a pre-test, (4) collecting data and (5) analysing the discrete choice data.

Identifying relevant preference attributes and levels is the key to design any stated-preference study [33]. People take various issues into account when deciding where they would choose to receive healthcare services. The chosen attributes and levels should be realistic and credible to respondents, so we considered two complementary stages to determine the attributes: literature review and expert consultation.

Evidence from previous research provides insights into public responses to choices, the literature review suggests the following list of ten attributes as most useful to be included: the established reputation, the opening hours [34], the follow-up arrangements [35], the type of insurance [36], the accessible of information [37], the shared decision making [38], the location of first appointment [39], the travel distance [40], the care provider [41]. Considering China’s context, the mode of services was included as a specific attribute.

We conducted expert consultations in December 2017 in Wuhan, China, aiming to understand the population’s current views on which factors are the most influential when choosing the healthcare service. Attributes from the literature review were collected to produce one manageable and comprehensive list for experts to rank, and ten experts (four scholars in health management and policy, three medical experts, three primary-level medical practitioners) were invited. Each participant was asked to individually rank the ten attributes in order of one (most important) to ten (least important) using a ranking sheet. Individual rankings were transferred into an Excel sheet for analysis. The included attributes should rank within the top five by ⩾60% of the experts. Whilst the literature is inconclusive with regard to the number of attributes that should be included in DCE, a review shows that most studies include 4–6 attributes [42]. Based on ranking results, we chose four attributes that are most relevant to policymaking, including (1) care provider;(2) mode of services;(3) cost;(4) travel time. The care provider is the professional staff who are responsible for providing diagnosis and treatment for resident’s healthcare demand including general practitioner (GP) and expert. The mode of services is the way of diagnosis and treatment including Traditional Chinese medicine (TCM), western medicine (WM) and integrated TCM and WM. The travel time refers to the time from home to healthcare institutions by the most convenient transportation way including ≤30 mins and >30 mins.

The next step was to refine the terminology that described the attributes and their levels. The attributes were designed at 2 or 3 levels each, capturing a realistic range within China’s healthcare system. For example, based on the 2017 China Statistical Yearbook, the cost ranged from CNY100 to 300 ($14 to 43). We presented all choices to our convenience sample in three formats (pictures, written description, and oral explanation). We found that a combination of written description and oral explanation was more reliable because this form would help respondents understand clearly. Table 1 presents the final attributes and levels used in our DCE.

**Table 1:** Attributes and levels used in the DCE

Attribute	Definition	Level
Care provider	The professional staff who is responsible for providing diagnosis and treatment for resident’s healthcare demand	1.Expert
		2.General practitioner (GP)
Mode of services	The way of diagnosis and treatment	1.Traditional Chinese medicine (TCM)
		2.Western medicine (WM)
		3.Integrated TCM and WM
Cost	Amount of money paid by patient (CNY)	1.CNY100 ($14) per time
		2.CNY200 ($29) per time
		3.CNY 300 ($44) per time
Travel time	Time from home to health institutions by the most convenient transportation way	1. < =30 mins
		2. > 30 mins

### Questionnaire design

The combination of attributes and levels resulted in 36 possible scenarios (two attributes at two levels and two attributes at three levels = 2^2^*3^2^). This was a heavy burden on respondents that could adversely affect response rates. An orthogonal design using SPSS software (version 22.0) was conducted to reduce the number of choice scenarios to a practical number of 16 representative combinations, which were further divided into two blocks to reduce the burden of respondents. The two versions of questionnaires were randomly allocated to respondents. The two scenarios consisting of the four attributes were labelled as Screening Option 1 and Screening Option 2, respectively (A sample scenario is shown in Table 2). We did not leave respondents an opt-out option. This is consistent with our experiment setting: the respondents are assumed to receive medical service and must choose the service content. Moreover, an opt-out only introduces slight differences into the estimations, [43] whereas the forced-choice method leads to more thoughtful responses and better-quality data [44].

**Table 2:** Example of a choice scenario

Attributes	Screening Option 1	Screening Option 2
Mode of services	Integrated TCM and WM	TCM
Cost	100 CNY	200 CNY
Time travelled to medical institution	≤30 min	>30 min
Care provider	Expert	GP
**Which one would you prefer?**	□	□

In addition to the DCE choices, the survey collected information on respondent’s socio-demographic characteristics (e.g. sex, age, marital status, education, occupation and household size, personal monthly income) and current health situation (health insurance coverage, number of diagnosed chronic diseases). The above information was used to assess the representativeness of respondents, as well as to use as predictive factors of class membership for the analysis.

The questionnaire itself was structured as follows. First, the background of the survey was briefly introduced, followed by the 12 demographic questions. Subsequently, a warm-up question was provided to the respondents that explained the layout of the questionnaire, and then we provided people with a rationality test. Given that the respondents had an a priori tendency toward attribute level ordering, we made one of the choice alternatives clearly superior. Participants who did not choose the dominant alternative were considered to fail the test [45] and were excluded from this study. Finally, pairwise DCE choice tasks were shown. All the questionnaire information can be seen in Additional files 1 and 2.

### Pilot testing

To check the respondents’ completion rates and the understanding of the questionnaire, a pilot study was undertaken among 60 voluntary residents in a community in Wuhan, China. Respondents needed 16.3 min on average to complete the questionnaire. We made minor changes to the layout, and our questionnaire was thought to be appropriate in length and understood easily by respondents through the pilot study.

### Data collection

To make the sample representative, we selected six cities in the eastern, central and western region of China, namely, Pudong New Area, Taizhou, Wuhan, Xuchang, Chengdu and Guiyang. In each city, we selected 1 urban district and 1 suburban district randomly. In each district, we selected 3 primary care facilities according to economic level. In total, 36 primary care facilities were chosen.

We followed the rule of thumb proposed by Pearmain [46] that sample sizes over 100 are able to provide a basis for modelling preference data in DCE design, therefore we selected a sufficient sample size of 60 participants from each facility. A stratified sampling method was used to ensure the sample representativeness. Specifically, the strata were pre-defined by gender (female or male) and age (18–45 years or > 45 years) of the local population [47].

After deciding the sampling sites and sample size, we cooperated with the 6 local government departments. The survey was supported by the local health bureau, and the research results would be provided to the bureau. Then the local health bureau notified the directors of primary healthcare facilities to assist in our survey preparation and organization. The directors of the sampled healthcare facilities were very cooperative and well prepared, they first screened the residential registration databases to find eligible respondents according to the given strata of gender and age, and the eligible respondents were permanent residents lived in the district including non-registered residents living in this area for more than half a year. Then the respondents were contacted to participate in the subsequent survey through phone or door-to-door visits, until the pre-defined sample quota was reached.

A formal survey was conducted in facilities. Each sampled primary healthcare facility organized a free physical examination for the respondents. After finishing the examination, the respondents took part in a face-to-face interview conducted by our trained interviewers, and paper-based questionnaires were used to ensure access and a good response rate. Informed consent of respondents was required before conducting the survey, and all of the respondents’ information remained anonymous.

The interviewer specified the context and the objective of the study, the meaning of the attributes and their levels and presented an example of a choice set. During the survey, the interviewer observed the respondents’ behaviour. If the choice was made based on a single attribute only [48], the respondent will be regarded as ineligible. When the surveys were finished, gifts (approximately 7.5 US dollars) were rewarded for the respondents. The inclusion criteria required the respondents who are over 18 years old, have had utilized medical services previously, could understand Chinese and independently complete the questionnaire. All documents involved in conducting the study were approved by the Ethics Committee of Tongji Medical College. This cross-sectional study was conducted from April to August 2018. In total, 2160 questionnaires were collected, and 2019 respondents passed the rationality test.

### Statistical analyses

The choice data are analysed within a random utility maximization framework [49]. The most common analysis method is the use of mixed logit model to quantify the relative importance of each attribute and another one is the latent class analysis (LCA).

Considering that the population is heterogeneous in its health status owing to the variability in how health dimensions manifest over time [50] and that the DCE also contains multiple dimensions, our approach embraces this heterogeneity and complexity. Response data were analysed using the LCA, where consideration is given to both the importance of the four DCE attributes for the different classes, as well as determinants of class membership, socio-demographic factors used to predict membership were: sex (measured as female and male), age and education (in years), whether they had basic medical insurance and were under the hypothetical scenario (minor or severe chronic diseases). In this paper, the selection of the optimal number of classes was guided by the Akaike Information Criterion (AIC), the Bayesian Information Criterion (BIC), the LMR (Lo-Mendell-Rubin) test, and the entropy. All models were specified and estimated in R-3.4.4. software.

## Results

### Description of sample

A total of 2019 valid respondents participated in this study, including 659 men and 1360 women; 47.69% of respondents were within the 18–44 age group. More than 70% of respondents received more than 9-years education and the majority of respondents were married and employed. 92.11% of respondents were covered by basic medical insurance. Self-reported results indicated that 71.93% had no chronic diseases. The mean monthly income was CNY 4415.31 (4415.31 ± 465.34) or ($641.79 ± 67.59). Table 3 illustrates the profile of the sample.

**Table 3:** The characteristics of 2019 respondents

	Full sample	Analysis sample: Who passed the rationality test	Excluded sample: Who failed the rationality test	χ^2^(*Ρ*-value)
	*n* = 2160	*n* = 2019	*n* = 141	
Variables	n (%)	n (%)	n (%)	
Sex				
Male	734(33.98)	659(32.63)	75(53.19)	2.37(.158)
Female	1426(66.02)	1360(67.37)	66 (46.81)	
Age (years)				1.65(.343)
18–44	981(45.41)	963(47.69)	18(12.76)	
45–59	495(22.92)	448(22.18)	47(33.33)	
≥ 60	684(31.67)	608 (30.13)	76(53.91)	
Marital status				2.11(.145)
Married	1767(81.81)	1700(84.20)	67(47.52)	
Unmarried	260(12.03)	211(10.45)	49(34.75)	
Divorced/Separated/Widowed	133(6.16)	108(5.35)	25(17.73)	
Length of education (years)				4.98(.274)
<9	496(22.96)	432(21.39)	64(45.39)	
9–15	908(42.03)	884(43.78)	24(17.02)	
>15	756(35.01)	703(34.83)	53(37.59)	
Monthly income (CNY)				1.67(.312)
≤ 3499	1003(46.43)	961(47.59)	42(29.78)	
3500-6000	720(33.33)	669(33.13)	51(36.17)	
>6000	437(20.24)	389(19.28)	48(34.05)	
Employment				1.35(.659)
Employed/working	1109(51.34)	1071(53.04)	38(26.96)	
Unemployed	535(24.76)	471 (23.32)	64(45.39)	
Retired	516(23.90)	477(23.64)	39(27.65)	
Health insurance				0.83(.846)
Insured	1971(91.25)	1857(92.11)	114(80.85)	
Uninsured	189(8.75)	162(7.89)	27(19.15)	
Chronic conditions				0.76(.753)
Yes	651(30.14)	566(28.07)	85(60.28)	
No	1509(69.86)	1453(71.93)	56(39.72)	
Age (${\overline{x}}\pm s$, years)	47.50 ± 18.11			
Monthly income ($\overline{x}\pm s$, CNY)	4415.31 ± 465.34			

### Patterns of similar value of health care utilization attributes

Table 4 displays the results of fitting indices of competing latent class models (two to five classes). As the number of classifications increased, both AIC and BIC initially increased and then decreased. When classification was 4, these two indicators were the lowest and the value of entropy was highest. Meanwhile, the LMR test that compared the three-class versus the four-class model achieved a significant level (*P* < 0.01). In addition, the LMR test that compared the four-class versus the five-class model did not achieve a significant level (*P* > 0.05), suggesting that the four-class model was the best one.

**Table 4:** Model fit information for competing latent class model

Number of classes	Log(L)	AIC	BIC	entropy	LMR
2	−19,913	39,877.72	40,095.68	0.363	0.241
3	−19,824	41,049.00	41,434.62	0.389	0.461
4	−19,657	39,445.25	39,999.03	0.433	< 0.01
5	−19,621	39,440.75	40,156.19	0.402	0.453

The results of the optimal model with four classes are given in Table 5: the upper part shows the regression coefficients for the four attributes of each class and the lower part shows the association of each class with demographic variables.

**Table 5:** Results of parameter estimation of LCA

Preference	Group 1			Group 2			Group 3			Group 4		
	Coefficient	SE.	*Z*-value	Coefficient	SE.	*Z*-value	Coefficient	SE	Z value	Coefficient	SE	*Z*-value
**Utility function**												
TCM^a^	−0.4977^***^	0.0735	**−**6.7624	**−**0.4064	1.1446	**−** 0.3551	5.3396	5.2419	1.0181	−0.5935^***^	0.0574	−10.3316
WM^b^	– 0.0148	0.059	**−**0.2506	1.6497	1.1291	1.4612	3.9783	2.7063	1.4700	**−**0.0326	0.0531	−0.6136
Cost	0.0034^***^	0.0009	3.4823	−0.0809^***^	0.0163	**−**4.9428	0.0359	0.0350	1.0277	**−**0.0008	0.0005	−1.6432
Travel time^c^	−0.611^***^	0.0675	**−**9.0253	0.1395	0.2421	0.5765	−8.5006^*^	4.0320	**−**2.1083	0.4658^***^	0.0495	9.4014
Provider^d^	−0.3681^***^	0.0956	**−**3.8476	0.1035	0.4129	0.2507	**−**1.8776	1.3905	**−**1.3503	1.4272^***^	0.0755	18.8857
**Influence factors**												
Sex^e^				**−**0.0531	0.0824	**−** 0.6432	**−**0.3077	0.2814	**−**1.0934	−0.0463^*^	0.1843	−2.5165
Age				−0.0078^*^	0.0039	**−**2.0104	−0.0625^***^	0.0131	**−**4.7918	0.0051^***^	0.0089	−5.6311
Education^f^				−0.2644^***^	0.0667	**−**3.9647	**−**0.2986	0.2035	**−**1.4672	**−**0.1666	0.1397	−1.1932
Insurance^g^				0.1496	0.1377	1.0862	4.8480	4.8685	0.9958	0.9840^***^	0.2904	3.3877
Severity^h^				0.6134^*^	0.2713	2.3266	3.1523^***^	0.4931	6.3928	4.3671^***^	0.4465	9.7792
**Class share**	N	%		N	%		N	%		N	%	
Predicted membership	551	27.29		763	37.79		15	0.74		690	34.18	

Note: In this section, only the significant values are highlighted in bold. The cost and age are continuity variables

^*^p < 0.05; ^**^p < 0.01; ^***^p < 0.001

^a^Compared with western medicine

^b^Compared with integrated TCM and WM

^c^Compared with less than or equal to 30mins

^d^Compared with GP

^e^compared with male

^f^Compared with shorter length of education(≤9 years)

^g^Compared without basic medical insurance

^h^Compared with the assumption that have minor chronic disease

A label is assigned to each profile based on comparisons of attribute probabilities, which are shown as follows:

The first class (*n* = 551,27.29%) was characterised by high probabilities of choosing all four attributes, indicating that this group had an even preference for each attribute and could obtain utility from the improvement of medical services. We labelled this group of people as “Comprehensive consideration”.

The second class was the largest group (*n* = 763, 37.79%) and named as “Price-driven”, with relatively high probabilities of choosing low prices, because only the coefficient of cost was significantly negative. Compared with the other three groups, the coefficient of medical expenses had a significantly higher level, indicating that this group valued price more highly than other attributes.

The third class (*n* = 15,0.74%) was labelled as “Near distance”: only the attribute of travel time was statistically negative significant, the coefficients of attributes and demographic factor jointly showed the profile of the respondents. They were younger people under the assumption in severe chronic disease and had a clear preference for near distance. This group was small but had a clear preference for seeking healthcare services nearby, suggesting that distance influence the preferences of some people, but not on a large scale.

In the last group (*n* = 690,34.18%), individuals had the highest probabilities of choosing experts, the utility coefficient of the care provider was positive and the absolute values were significantly higher than those of the other three attributes. The coefficient of the attributes of distance and medical expenses were smaller numerically than others. Therefore, we labelled this group “Quality seeker”.

The influence of demographic factors showed that individuals with lower education and younger ages had a high probability to enter group 2, people who were males, older, insured and assumed to have severe chronic diseases were more likely to enter group 4, but younger people who assumed to have severe chronic diseases were more likely to enter group 2.

### Measuring willingness to pay

WTP represents the mean maximum monetary equivalent of improvement in a single attribute and level [51], we can compute the ratio between the coefficient for each attribute and the price coefficient [52] to calculate the WTP. The results are shown in Table 6.

**Table 6:** Results of WTP for each attribute

Level	Gourp1	Group2	Group3	Group4	Average (95% CI)
Mode of services	144.68	−5.02	− 148.36	697.78	−202.12(− 204.43 to − 201.34)
Cost	4.32	20.37	110.58	− 38.34	+ 4.90(4.63 to 7.36)
Travel time to medical institution	177.51	1.73	236.29	547.62	+ 238.01(222.48 to 244.83)
Care provider	107.04	1.28	52.19	1677.85	+ 604.31(591.76 to 610.45)

The average WTP showed that respondents’ value of different attributes in healthcare usage from high to low was: care provider, travel time, mode of services. The quality seekers were willing to pay more for better service instead of settling for other factors, which supports the notion that this group prioritizes medical service quality.

The positive (+) /negative (−) results indicate theoretically to what extent the respondents would be willing to pay for or be compensated for an attribute level. The individuals were willing to pay CNY 604.31($87.84) to be treated by experts. A subsidy of CNY 202.12($29.37) was required for respondents to accept more TCM services.

## Discussion

To the best of our knowledge, this is the first study to use the latent class analysis to identify a typology of preferences for health care usage across multiple dimensions in China. The LCA reveals four distinct patterns in health care choices and characteristics. Respondents can be divided into four groups: comprehensive consideration, price-driven, near distance and quality seeker. Different factors drive people to make their choice of healthcare usage, revealing preference heterogeneity.

The results of group 1 indicate that healthcare policy will need to be multidimensional. The preferences of respondents for multiple dimensions of health care usage suggests that the policy focus solely on strengthening medical capabilities might not be acceptable. Indeed, all respondents place value on travel time, suggesting that medical care accessibility and availability are important. We also find that the proportion of group 2 and 4 is high. From this perspective, they are more likely to be influenced by a broader influence of the allocation of healthcare resources.

Furthermore, respondents in group 2 would prefer primary healthcare services at lower prices, which is in line with the pursuit of maximizing utility. Moreover, in China, one of the government’s goals for developing the primary health care system is to provide residents with services that are relatively inexpensive and convenient. According to the results of our study, the goal of this policy is consistent with the residents’ preferences.

Interestingly, choosing to visit a large-size hospital or medical expert has been the level the respondents in group 4 preferred most for care, regardless of traveling distance. Many residents in China still go to hospitals for both minor and serious illnesses [53]. One of the most important reasons is that residents seek healthcare unrestrictedly. A notion has been held in Chinese is that a higher level of health institutions provides better quality care. The past imbalanced allocation of health resources has intensified residents’ distrust of primary healthcare facilities. Hence, the tiered medical system would not be successful if residents’ opinions are ignored. To address this complicated problem, it is necessary to guide residents to seek medical treatment based on their perceived value for service quality [54] and carry out comprehensive health-centred services in primary healthcare facilities.

A unique result is that the respondents of group 3 have a clear and significant preference for near distance. Travelling distance to healthcare facilities will affect patients’ medical decision-making by considering time and transportation cost. It is more likely that due to the increase in the degree of information asymmetry, patients will appear irrational behaviours of “loss avoidance” preferences in uncertain environments [55]. The presence of distance decay effects in healthcare services has been demonstrated by most studies [56]. A review shows that the further a patient lives from the healthcare facilities they need to attend, the worse their health outcomes are [57]. These suggest that distance/travel time should be a consideration when configuring the health resources. China is trying to establish a 15-min circle of healthcare services by the year 2030 and residents can walk within 15 min to the most convenient medical centers around their home. The convenience of healthcare service has been emphasized and by combining such factors as distance, accessibility will ensure the equity of healthcare services.

Our large-scale study also provides new information about demographic characteristics and health status that could have a significant impact on healthcare preferences. Men, the elderly, the insured, and those who assumed to have severe chronic diseases will pay more attention to service quality. People with lower education and younger age would pay more attention to the price of medical services. We can find that the quality and price of healthcare service are the core of basic healthcare and the cornerstone of the desired medical and health service system. With an ageing population and an increase in chronic diseases and comorbidities, health services should concentrate on the health of the population and devote attention to the community residents to prevent disease, decrease prevalence or delay its onset [58]. Previous studies have shown that low-income people are not only in a disadvantaged position in wealth, but also in health [59]. Therefore, we should consider subsidising medical care for low-income people to improve their health. On the other hand, we can strengthen prevention by expanding health promotion and education to reduce the likelihood of illness.

There are great differences among respondents who are willing to pay for healthcare services. Results show that people have the highest level of the willingness to pay for expert services, reflecting that the public values medical service quality. On the other hand, our research finds that a subsidy of CNY 202.12($29.37) is important for respondents to accept more TCM services. It indicates that the level of compensation should be in line with public preferences to improve the benefit and accessibility of primary health services. The TCM is profound, has its own unique advantages and can be conducive to provide better personalized and targeted primary healthcare services. The vigorous development of traditional medicine services is inseparable from policy support, which can achieve the goal of optimising medical resource allocation and harmonising the diversity of healthcare demand. It is necessary to formulate the supporting policy such as increasing human resources and technical support, which will bring TCM’s superiority into full play in primary healthcare facilities [60].

Health service usage is a complex behaviour affected by many factors, and the preferences of individuals may change as a function of one’s health status [61]. More research is needed to examine these groups in different contexts. One is interactions between attributes and their effects on preference for healthcare usage. Given the complex and multifaceted nature of health, it may be that preferences for one attribute are influenced by preferences for another. Another is to examine factors that lead these groups to map future transition of service use, deep qualitative studies about quality of life over time or large cohort studies need to be conducted. This would help policymakers and healthcare providers align with public’s preferences. There are more women than men in our study, part of it is that gender imbalanced structure in older people has become a noticeable issue in China’s population distribution, and the statistical yearbook data in 2019 shows that there are more elderly women than men, with a sex ratio of less than 100 [62]. The other part is due to the hollowed-out of population structure in rural and suburban areas, the massive of young and middle-aged labour force flow outward, resulting in that most of the population in rural and suburb areas are elderly, women [63, 64]. In the actual survey, women were also more attracted by our incentives and they were more proactive and enthusiastic in taking part our study in the workday.

### Limitations

Several study limitations should be noted. First, the LCA can identify the preference heterogeneity, but are unable to discover the underlying behavior reason in depth, and can be better explained through qualitative interviews [65]. Second, since a maximum number of five attributes is suggested, we selected the most important attributes for the study. There may be some other factors that are missed. Third, respondents need to make choices under certain circumstances, and the stated preference may be different from the revealed preference. We used WTP merely as an estimate of the relative utility of services and not as an indication of true WTP [66]. Fourth, the probable cause for unbalanced gender structure has been explained above and this reminds us to deal with the balance between representativeness of sample and research feasibility.

## Conclusions

Among people using healthcare services, we find that their behaviour could be described in four groups: overall consideration, price-driven, near distance and quality seeker. The results suggest that the care provider, mode of services, travel time and cost should be considered in priority setting decisions. The study also reveals substantial disagreement in the opinion of Traditional Chinese Medicine between different population subgroups. Effectively measuring the preference of healthcare delivery from the perspective of the demander is essential to increase service efficiency within resource constraints. The policy should be multidimensional, not only emphasis strengthening the accessibility and availability of healthcare services, but also guide the residents to seek healthcare services properly and rationally. Moreover, the policy should consider improving the socio-economic status of the population to improve their health outcome.

## Supplementary information

**Additional file 1.** *Questionnaire: Version 1.

**Additional file 2.** * Questionnaire: Version 2.

## Data Availability

The datasets used and analyzed during the current study are available from the corresponding author on reasonable request. Contact information: xiongjuyang@hust.edu.cn.

## Abbreviations

LCA: Latent class analysis
DCE: Discrete choice experiment
WTP: Willingness to pay
TCM: Traditional Chinese Medicine
GDP: Gross domestic product
LPCP: The London Patient Choice Project
GP: General practitioner
WM: Western medicine
AIC: Akaike information criterion
BIC: Bayesian information criterion
LMR: Lo-Mendell-Rubin test
