# Supplementary material for: Preferences heterogeneity of health care utilization of community residents in China: a stated preference discrete choice experiment
Source: BMC Health Serv Res. 2020 May 18;20:430. doi: 10.1186/s12913-020-05134-4 (PMC7236293; doi:10.1186/s12913-020-05134-4)
Supplement: Supplementary file 1 — Additional file 1. *Questionnaire: Version 1. [file 12913_2020_5134_MOESM1_ESM.docx]

**Part 1: Respondents’ socio-demographic characteristics**

***1. Age: ____***

***2. Gender:***

A: Male B: Female

***3. Marital status:***

A: Unmarried B: Married C: Divorced/Separated/Widowed

***4.*** ***Length of education(years):***

A: ＜9 B: 9-15 C:＞15

***5.Employment：***

A: Employed/working B: Unemployed C: Retired

***6. Monthly individual income (CNY):***

A: ≤3499 B: 3500-6000 C:> 6000

***7.Health insurance（multiple choices）***

A: Medical insurance for urban workers B: Medical insurance for urban residents

C: New rural cooperative medical insurance D: Commercial insurance E: No health insurance

***8. Household size：____***

***9. Whether diagnosed with a chronic disease?***

A: With chronic disease B: Without chronic disease

***10.Number of diagnosed chronic diseases*: ______**

***11.*** ***Is chronic illness put a heavy financial burden on your family?*** ***（if you have chronic disease）***

A:Very heavy and stressful B：Heavier and more stressful C: Not heavy

***12.*** ***What is your usual treatment for chronic diseases?*** ***（if you have chronic disease）***

A: Medical treatment B. Non-drug therapy

C. Combination of medication and non-drug therapy D. No intervention

**Part 2：The questionnaire of discrete choice experiments（DCE）**

Suppose you are in the physical state described in the following case. Under this premise, if you now have two options for health care utilization, option 1 and option 2. These two options differ in terms of mode, cost, time and care provider. Please choose the preferred option according to your personal preferences and tick the corresponding boxes. When you make choices, please assume that the conditions are the same except for the four attributes listed.

**Rationality test**

| Attributes | Screening Option 1 | Screening Option 2 |
| --- | --- | --- |
| Mode of services | Integrated TCM and WM | Integrated TCM and WM |
| Cost | 300 CNY | 100 CNY |
| Time travelled to medical institution | ＞30 min | ≤30 min |
| Care provider | Expert | Expert |
| **Which one would you prefer?** | □ | □ |

Suppose you have a minor chronic disease that occasionally makes you feel uncomfortable and does not seriously affect daily life, even without going to a doctor. Please make your preferred choice.

**Project 1**

| Attributes | Screening Option 1 | Screening Option 2 |
| --- | --- | --- |
| Mode of services | WM | Integrated TCM and WM |
| Cost | 300 CNY | 100 CNY |
| Time travelled to medical institution | ≥30 min | ＜30 min |
| Care provider | Expert | GP |
| **Which one would you prefer?** | □ | □ |

**Project 2**

| Attributes | Screening Option 1 | Screening Option 2 |
| --- | --- | --- |
| Mode of services | TCM | WM |
| Cost | 200 CNY | 300 CNY |
| Time travelled to medical institution | ≤30 min | ＞30 min |
| Care provider | GP | Expert |
| **Which one would you prefer?** | □ | □ |

**Project 3**

| Attributes | Screening Option 1 | Screening Option 2 |
| --- | --- | --- |
| Mode of services | WM | Integrated TCM and WM |
| Cost | 100 CNY | 200 CNY |
| Time travelled to medical institution | ≤30 min | ＞30 min |
| Care provider | GP | Expert |
| **Which one would you prefer?** | □ | □ |

**Project 4**

| Attributes | Screening Option 1 | Screening Option 2 |
| --- | --- | --- |
| Mode of services | Integrated TCM and WM | WM |
| Cost | 300 CNY | 100 CNY |
| Time travelled to medical institution | ≤30 min | ＞30 min |
| Care provider | GP | Expert |
| **Which one would you prefer?** | □ | □ |

**Project 5**

| Attributes | Screening Option 1 | Screening Option 2 |
| --- | --- | --- |
| Mode of services | Integrated TCM and WM | WM |
| Cost | 300 CNY | 100 CNY |
| Time travelled to medical institution | ≤30 min | ＞30 min |
| Care provider | GP | Expert |
| **Which one would you prefer?** | □ | □ |

**Project 6**

| Attributes | Screening Option 1 | Screening Option 2 |
| --- | --- | --- |
| Mode of services | WM | Integrated TCM and WM |
| Cost | 100 CNY | 200 CNY |
| Time travelled to medical institution | ＞30 min | ≤30 min |
| Care provider | GP | Expert |
| **Which one would you prefer?** | □ | □ |

**Project 7**

| Attributes | Screening Option 1 | Screening Option 2 |
| --- | --- | --- |
| Mode of services | TCM | WM |
| Cost | 200 CNY | 300 CNY |
| Time travelled to medical institution | ＞30 min | ≤30 min |
| Care provider | Expert | GP |
| **Which one would you prefer?** | □ | □ |

**Project 8**

| Attributes | Screening Option 1 | Screening Option 2 |
| --- | --- | --- |
| Mode of services | Integrated TCM and WM | TCM |
| Cost | 100 CNY | 200 CNY |
| Time travelled to medical institution | ＞30 min | ≤30 min |
| Care provider | Expert | GP |
| **Which one would you prefer?** | □ | □ |

Suppose you have a serious chronic disease that seriously affect your daily life. After seeing a doctor, you can extend the survival time and improve the quality of life, but the disease may not be cured. Please make your preferred choice.

**Project 1**

| Attributes | Screening Option 1 | Screening Option 2 |
| --- | --- | --- |
| Mode of services | WM | Integrated TCM and WM |
| Cost | 300 CNY | 100 CNY |
| Time travelled to medical institution | ≥30 min | ＜30 min |
| Care provider | Expert | GP |
| **Which one would you prefer?** | □ | □ |

**Project 2**

| Attributes | Screening Option 1 | Screening Option 2 |
| --- | --- | --- |
| Mode of services | TCM | WM |
| Cost | 200 CNY | 300 CNY |
| Time travelled to medical institution | ≤30 min | ＞30 min |
| Care provider | GP | Expert |
| **Which one would you prefer?** | □ | □ |

**Project 3**

| Attributes | Screening Option 1 | Screening Option 2 |
| --- | --- | --- |
| Mode of services | WM | Integrated TCM and WM |
| Cost | 100 CNY | 200 CNY |
| Time travelled to medical institution | ≤30 min | ＞30 min |
| Care provider | GP | Expert |
| **Which one would you prefer?** | □ | □ |

**Project 4**

| Attributes | Screening Option 1 | Screening Option 2 |
| --- | --- | --- |
| Mode of services | Integrated TCM and WM | WM |
| Cost | 300 CNY | 100 CNY |
| Time travelled to medical institution | ≤30 min | ＞30 min |
| Care provider | GP | Expert |
| **Which one would you prefer?** | □ | □ |

**Project 5**

| Attributes | Screening Option 1 | Screening Option 2 |
| --- | --- | --- |
| Mode of services | Integrated TCM and WM | WM |
| Cost | 300 CNY | 100 CNY |
| Time travelled to medical institution | ≤30 min | ＞30 min |
| Care provider | GP | Expert |
| **Which one would you prefer?** | □ | □ |

**Project 6**

| Attributes | Screening Option 1 | Screening Option 2 |
| --- | --- | --- |
| Mode of services | WM | Integrated TCM and WM |
| Cost | 100 CNY | 200 CNY |
| Time travelled to medical institution | ＞30 min | ≤30 min |
| Care provider | GP | Expert |
| **Which one would you prefer?** | □ | □ |

**Project 7**

| Attributes | Screening Option 1 | Screening Option 2 |
| --- | --- | --- |
| Mode of services | TCM | WM |
| Cost | 200 CNY | 300 CNY |
| Time travelled to medical institution | ＞30 min | ≤30 min |
| Care provider | Expert | GP |
| **Which one would you prefer?** | □ | □ |

**Project 8**

| Attributes | Screening Option 1 | Screening Option 2 |
| --- | --- | --- |
| Mode of services | Integrated TCM and WM | TCM |
| Cost | 100 CNY | 200 CNY |
| Time travelled to medical institution | ＞30 min | ≤30 min |
| Care provider | Expert | GP |
| **Which one would you prefer?** | □ | □ |
